# Short‐term physiological plasticity: Trade‐off between drought and recovery responses in three Mediterranean *Cistus* species

**DOI:** 10.1002/ece3.3484

**Published:** 2017-11-12

**Authors:** Giacomo Puglielli, Rosangela Catoni, Alessandra Spoletini, Laura Varone, Loretta Gratani

**Affiliations:** ^1^ Department of Environmental Biology Sapienza University of Rome P.le Aldo Moro 5 00185 Rome Italy

**Keywords:** gas exchange, local adaptations, phenotypic plasticity, resilience

## Abstract

Short‐term physiological plasticity allows plants to thrive in highly variable environments such as the Mediterranean ecosystems. In such context, plants that maximize physiological performance under favorable conditions, such as *Cistus* spp., are generally reported to have a great cost in terms of plasticity (i.e., a high short‐term physiological plasticity) due to the severe reduction of physiological performance when stress factors occur. However, *Cistus* spp. also show a noticeable resilience ability in response to stress factors. We hypothesized that in *Cistus* species the short‐term physiological response to stress and that to subsequent recovery can show a positive trade‐off to offset the costs of the photosynthetic decline under drought. Gas exchange, chlorophyll fluorescence, and water relations were measured in *C. salvifolius*,* C. monspeliensis,* and *C. creticus* subsp*. eriocephalus* during an imposed experimental drought and subsequent recovery. Plants were grown outdoor in common garden conditions from seeds of different provenances. The short‐term physiological response to stress and that to recovery were quantified via phenotypic plasticity index (PI_stress_ and PI_recovery_, respectively). A linear regression analysis was used to identify the hypothesized trade‐off PI_stress_–PI_recovery_. Accordingly, we found a positive trade‐off between PI_stress_ and PI_recovery,_ which was consistent across species and provenances. This result contributes in explaining the profit, more than the cost, of a higher physiological plasticity in response to short‐term stress imposition for *Cistus* spp because the costs of a higher PI_stress_ are payed back by an as much higher PI_recovery_. The absence of leaf shedding during short‐term drought supports this view. The trade‐off well described the relative variations of gas exchange and water relation parameters. Moreover, the results were in accordance with the ecology of this species and provide the first evidence of a consistent trade‐off between the short‐term physiological responses to drought and recovery phases in Mediterranean species.

## 1 INTRODUCTION

As the rate of climate change is increasing, it might occur faster than the capacity of plant species to adapt (Ramírez‐Valiente, Valladares, Gil, & Aranda, 2009). Phenotypic plasticity rather than genetic diversity is likely to play a crucial role in allowing short‐term ecological response of plant species under rapid climate change (Gratani, 2014; Gratani et al., 2014; Puglielli, Crescente, Frattaroli, & Gratani, 2015; Vitasse, et al., 2010). Plasticity may even be adaptive, promoting species establishment and persistence under new climatic conditions (Ghalambor, McKay, Carroll, & Reznick, 2007; Nicotra, et al., 2010). However, some studies show that different environmental conditions can also drive to canalization processes (e.g., Lamy, et al., 2011; Valladares et al., 2002).

Rapid climate change represents a selective force that can drive genetic changes or induce phenotypic plasticity within populations (Jump & Peñuelas, 2005). However, in drought situations, phenotypic plasticity can succeed over evolutionary adaptation (Gimeno, Pías, Lemos‐Filho, & Valladares, 2009; Nicotra, Hermes, Jones, & Schlichting, 2007), even blurring ecotypic divergence (Baquedano, Valladares, & Castillo, 2008; Santiso & Retuerto, 2017).

Among the plethora of plastic responses that plant species can exhibit in response to drought, short‐term physiological plasticity in traits such as stomatal conductance and photosynthetic rate might be advantageous (Sultan, 2000). Drought represents one of the main limiting factors in Mediterranean ecosystems (Flexas et al., 2014) severely limiting the capacity for CO_2_ assimilation (Pastenes, Pimentel, & Lillo, 2005; Puglielli, Redondo‐Gómez, Gratani, & Mateos‐Naranjo, 2017). However, the plant capacity to face drought stress may depend as much as on the photosynthetic recovery (Varone & Gratani, 2015), which in turn depends on the degree and velocity of photosynthesis decline during water depletion (Chaves, Flexas, & Pinheiro, 2009). Such responses rely on short‐term physiological plasticity to contrasting environmental conditions.

Short‐term physiological plasticity is particularly important for widely distributed Mediterranean plant species, which represent a good model to investigate whether different provenances respond differently to water stress and recovery due to their short‐term physiological plasticity (Santiso & Retuerto, 2017).

Mediterranean *Cistus* spp. show a relatively high physiological plasticity in response to drought (Bongers et al., 2017; Catoni, Gratani, & Varone, 2012; Grant et al., 2014) due to their ability to maximize growth and physiological performance during the favorable periods (Bongers et al., 2017; Puglielli, Spoletini, Fabrini, & Gratani, 2017). Accordingly, a negative trade‐off between plant maximum photosynthetic rate in the favorable period and the ability to maintain photosynthesis under adverse conditions has been recently demonstrated (Zhang, Sack, Cao, Wei, & Li, 2017). However, *Cistus* spp. also show a noticeable resilience in their ability to respond to stress factors (see Correia & Ascensão, 2017 for a synthesis; Puglielli, Cuevas Román, et al., 2017).

We therefore hypothesized that the short‐term physiological response to stress and that to subsequent recovery, quantified via phenotypic plasticity index (PI, *sensu* Valladares et al., 2000), can show a positive trade‐off in order to offset the costs of the photosynthetic decline under adverse conditions. We tested our hypothesis in the three widespread *Cistus* spp. (namely *C*. *creticus* subsp. *eriocephalus*,* C*. *monspeliensis,* and *C*. *salvifolius*) grown from seeds of different provenances under common garden conditions. In particular, we asked the following questions: (1) Can such trade‐off be independent of the species and their strategies to cope with drought? (2) Can different provenances of the same species differ in terms of PI thus altering the expected trade‐off?

Such evidences are particularly important for *Cistus* spp., considering that they are representative of the early successional stages in the Mediterranean Basin shrublands (Puglielli, Varone, Gratani, & Catoni, 2017). Shrubs’ potential resilience to environmental changes deserves particular attention in those areas characterized by pronounced seasonal dynamics and threatened by climate change, such as the Mediterranean ones.

## 2 MATERIALS AND METHODS

### 2.1 Plant material, study area, and experimental conditions

Seeds of *Cistus monspeliensis* (CM), *C. salvifolius* (CS), and *C. creticus* subsp. *eriocephalus* (CE) from different provenances (*n* = 100 seeds per species) were obtained from Sardinian Germplasm Bank (BG‐SAR). In particular, *CM* seeds were collected in South Spain (referred as CMSp, Loc. Pantano Quebrajano, 37°37′57.7″N; 03°43′43.9″W, Andalucía) and in Sardinia (referred as CMS, Loc. Guspini, 39°32′32″N; 8°38′02″E, Medio Campidano). CS seeds were collected in southwest Spain (referred as CSSp, Loc. Huelva, 37°15′41″N; 6°57′54″W, Andalucia) and in Sardinia (referred as CSS, Loc. Portixeddu, 39°26′32″N; 8°24′37″E, Carbonia‐Iglesias). CE seeds were collected in Sardinia (referred as CES, Loc. Portixeddu, 39°26′32″N; 8°24′37″E, Carbonia‐Iglesias) and on the Italian mainland (referred as CEF, Loc. Foce del Garigliano, 41°13′23″N; 13°45′45″E, Caserta). The choice of CE seeds from the Italian mainland is because the species is not present in Spain. Details on seed germination protocols are reported in Puglielli, Spoletini et al. (2017).

The experiment was carried out under natural conditions inside the Botanical Garden of Sapienza University of Rome (41°53′33″N, 12°27′54″E, 17 m a.s.l.). For each species and provenance, two‐year‐old plants were selected. This plant age is important since the first 2 years after seedling establishment are critical for determining the successful recovery of *Cistus* spp. population in the natural environment (Quintana, Cruz, Fernández‐González, & Moreno, 2004).

Until the onset of the experiment (on 12 July 2016, Day 0), 90 plants (i.e., 3 species × 2 provenances × 15 individuals per provenance) were arranged in a completely randomized design (CRD) and watered regularly to field capacity. We then marked 10 shoots per plant within each provenance which were used for subsequent measurements.

Plant water status, gas exchange, and chlorophyll fluorescence measurements were performed on Day 0 when all the plants were well watered. Thereafter, the water stress was imposed by withholding water from ten plants from each provenance, and subsequent measurements were carried out every 3 days (namely Day 3, Day 6, Day 9 and Day 12).

The remaining five plants per provenance were kept under daily irrigation. The water stress experiment was stopped when stomatal conductance in stressed plants was below 0.05 mol m^−2^ s^−1^ which is indicative of a severe water stress condition (Medrano, Escalona, Bota, Gulías, & Flexas, 2002). After this threshold, irrigation was restored, and the recovery phase was followed. The recovery was considered to have occurred when stomatal conductance was above 0.20 mol m^−2^ s^−1^, which is a common value found for *Cistus* well‐watered plants in pot experiments (Gallé, Florez‐Sarasa, El Aououad, & Flexas, 2011; Puglielli, Redondo‐Gómez, et al., 2017). On the whole, the experiment lasted 12 days (the last day is hereafter referred as Day 12).

During the experiment, mean maximum daily air temperature was 34.5 ± 0.7°C, and mean minimum daily air temperature was 22.3 ± 2.1°C. Relative humidity varied between 18 and 56%. Data were obtained from the meteorological station placed in the Botanical Garden of Sapienza University of Rome.

### 2.2 Plant water status

Water potential (Ψ_Pd_, MPa) and relative water content (RWC_Pd_, %) were measured at predawn on three apical shoots belonging to different plants randomly selected from each provenance per sampling occasion. Ψ_Pd_ was measured by a pressure chamber (SKPM 1400, Sky Instruments, Powys, UK).

RWC_Pd_ was calculated as:

[(FW−DW)/(TW−DW)]×100,

where FW was the apical shoot fresh weight; DW was the dry weight after drying at 80°C until constant weight was reached; and TW was the apical shoot weight after rehydration until saturation in distilled water for 24 hr at 4°C in the darkness.

Being a pot experiment, and considering the below‐mentioned destructive measurements necessary to determine shoot gas exchange parameters, Ψ_Pd_, and RWC_Pd_ were only measured in the three main stages of the imposed stress: Day 0 (first day), Day 6 (maximum stress), and Day 12 (occurred recovery). This allowed us to limit excessive sampling of the available plant material.

### 2.3 Shoot gas exchange and chlorophyll fluorescence

Gas exchange measurements were performed using an infrared gas analyzer in an open system (LCpro+, ADC, UK), equipped with a 6.25 cm^2^ leaf chamber (PLC, Parkinson Leaf Chamber). Measurements were carried out in the morning (10.00–11.00 a.m.) on all the leaves of three to five apical shoots belonging to different plants and randomly selected from each provenance per sampling occasion. The following parameters were determined: net photosynthesis per unit leaf area (μmol CO_2_ m^−2^ s^−1^), stomatal conductance (mol H_2_O m^−2^ s^−1^), transpiration (mmol H_2_O m^−2^ s^−1^), and photosynthetic photon flux density (PPFD, μmol photons m^−2^ s^−1^). Gas exchange parameters were measured at ambient CO_2_ (of 400 ppm) and leaf temperature (coefficient of variation during measurements of 1%). Relative humidity ranged between 50 and 60% during measurements, and PPFD was always above 1,500.

Measurements of chlorophyll fluorescence were carried out on the same apical shoots of gas exchange measurements, using a portable modulated fluorometer (OS5p, Opti‐Sciences, USA).

Chlorophyll fluorescence measurements were carried out at midday at saturating PPFD (i.e., 1,500 μmol photons m^−2^ s^−1^) ensuring a uniform light distribution on leaf surface while maintaining an inclination of the fluorometer pulse source at 45°. Prior to photosynthesis measurements, maximum quantum efficiency of PSII photochemistry (*F*_v_/*F*_m_) was measured on all the leaves of each apical shoot of each plant after 30 min of dark adaptation with leaf clips. Basal fluorescence (*F*_0_) was obtained with a light pulse of ~0.1 μmol m^−2^ s^−1^, while maximum fluorescence (*F*_m_) with a light‐saturating pulse (~8,000 μmol m^−2^ s^−1^) for 0.8 s. *F*_v_/*F*_m_ was calculated as (*F*_m_–*F*_0_)/*F*_m_.

In each sampling occasion, after the completion of gas exchange and chlorophyll fluorescence measurements, the apical shoots were cut, and total leaf area (TLA, m^2^) was measured. The final dataset thus included net photosynthesis (*A*_a_, μmol CO_2_ s^−1^), stomatal conductance (*gs*, mol H_2_O s^−1^), and transpiration (*E*, mmol H_2_O s^−1^) for the entire apical shoot estimated by multiplying the mean value of each gas exchange parameter per TLA, while *F*_v_/*F*_m_ values per each apical shoot were obtained by averaging the *F*_v_/*F*_m_ measured for all the leaves of each apical shoot. The choice to express gas exchange parameters normalized by TLA, or as average for the entire shoot in the case of *F*_v_/*F*_m_, was due to the necessity to exclude any difference in leaf number between species and provenances. In order to detect eventual leaf shedding, which is a common response of *Cistus* spp. under drought (Correia & Ascensão, 2017), the total number of leaves per shoot was counted in both stressed and control plants on 10 apical shoots from each provenance and per sampling occasion before physiological measurements.

### 2.4 Data analysis

Pearson pairwise correlation coefficients and regression analysis were used to identify bivariate relationships between the considered traits at *p *≤* *.05.

The phenotypic plasticity index (PI, Valladares et al., 2000) was calculated for each provenance to quantify the degree of phenotypic plasticity in response to stress (PI_stress_) and recovery (PI_recovery_) for each of the considered traits. In particular, PI was calculated as: the [(maximum mean – minimum mean)/maximum mean]. For PI_stress_, the numerator was the difference between the mean value of the trait measured for stressed plants at Day 0 (i.e., first day of the experiment) and at Day 6 (i.e., day of maximum stress), and the denominator was the mean value of the trait measured at Day 0.

PI_recovery_ was calculated by replacing the value of the trait measured at Day 0 with that measured at Day 12 (i.e., maximum recovery). Then, PI_recovery_ was regressed against PI_stress_ via linear regression analysis (*n* = 36). In order to prove the predictive ability of the generated linear model, it was cross‐validated by randomly assigning the data to fivefolds (*n* = 7–8 per fold). Each fold was removed, in turn, while the remaining data were used to refit the regression model and to predict at the deleted observations. Cross‐validation was implemented through the function CV*lm* included in the R package *DAAG* (Maindonald & Braun, 2015).

Differences between provenances in PI_stress_ and PI_recovery_ were tested by one‐way ANOVA. Differences were considered significant at *p *≤* *.05. One‐way ANOVA was also used to test for within treatment differences in total leaf number per shoot between sampling days.

Moreover, to test if species shared a common pattern of drought stress response, a principal component analysis (PCA) per species by including all the considered physiological variables grouped by water treatment was carried out. Then, a two‐way ANOVA per species on the PC explaining the highest proportion of variance (i.e., PC1) was performed in order to test the effect of the factors provenance (*Pr*), water treatment (*WT*), and their interaction (Pr*WT). Multiple comparisons were analyzed by a Tukey test. Such approach was used in order to reduce multiple testing, considering that the use of emerging collective properties (expressed by PC1) as primary variable allows for an equally robust approach (Giuliani, 2017). PCA and two‐way ANOVA were performed only on data of the day of maximum stress (Day 6) in order to evaluate if the factor provenance could affect PI estimates.

## 3 RESULTS

**Figure 1:**
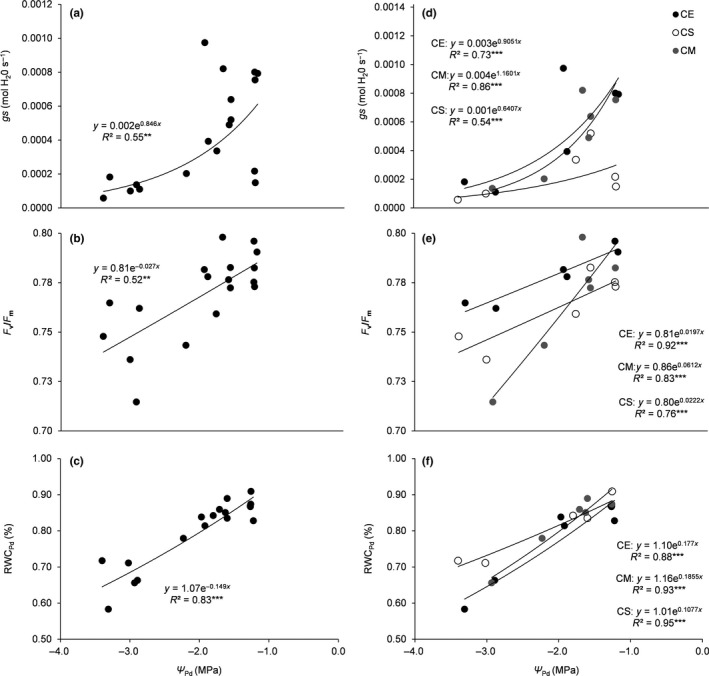
Relationship between predawn water potential (Ψ_Pd_) and (a) stomatal conductance (*gs*), (b) maximum quantum efficiency of PSII photochemistry (*F*_v_/*F*_m_), and (c) predawn relative water content (RWC_Pd_) across species and days of experiment (*n* = 3 stressed plants per provenance). The panels d, e, and f display the same relationships within each species. The fitted equations as well as their *R* ^2^ are shown. ** and *** indicate *p* < .01 and .0001, respectively. CE, *Cistus creticus* subs. *eriocephalus*; CS, *C. salvifolius,* and CM, *C. monspeliensis*

During stress imposition, Ψ_Pd_ decreased significantly reaching the lowest values at Day 6 for all the considered provenances, with values between −2.23 and −3.4 MPa. At Day 12, Ψ_Pd_ was on average 77% of that measured at Day 0. Pearson pairwise correlation coefficients were all significant highlighting a strong coordination among the physiological variables (mean *R* = 0.79 ± 0.13). *gs*,* F*_v_/*F*_m_, and RWC_Pd_ declined in parallel with Ψ_Pd_ and recovered in Day 12 (Figure 1a–c). These relationships also held at the intraspecific level, even if CE and CM showed higher *R* ^2^ for the relationships *gs*–Ψ_Pd_ and *F*_v_/*F*_m_–Ψ_Pd_ compared to CS. The latter, in turn, showed the highest *R* ^2^ for the relationship RWC_Pd_–Ψ_Pd_ (Figure 1d–f).

The PCA returned two axes of variation for each species with percentages of explained variance between 78 and 80 for PC1 and between 11 and 14 for PC2. PC1 was in fact correlated (*p* < .05) with all the physiological variables (Table 1), independently of the species and the provenance, highlighting a reduced performance in stressed plants compared to control ones. On the other hand, PC2 showed an eigenvalue lower than 1, so it was discarded from farther analyses.

**Table 1:** Factor loadings for principal component analysis (PCA) carried out on the considered physiological traits for *Cistus creticus* subs. *eriocephalus* (CE), *C. salvifolius* (CS), and *C. monspeliensis* (CM)

	CE		CS		CM	
	PC1	PC2	PC1	PC2	PC1	PC2
% Variance	80	14	78	11	79	13
*A*_a_	**0.98**	0.16	**0.97**	0.13	**0.98**	−0.11
*gs*	**0.94**	0.25	**0.93**	0.34	**0.98**	−0.11
*E*	**0.98**	0.15	**0.91**	0.38	**0.95**	−0.09
*F*_v_/*F*_m_	**0.80**	0.41	**0.74**	−**0.57**	**0.57**	**0.80**
Ψ_Pd_	−**0.87**	0.46	−**0.89**	0.33	−**0.87**	0.34
RWC_Pd_	**0.80**	−**0.60**	**0.87**	−0.07	**0.90**	0.17

*A*_a_, net photosynthesis per unit leaf area; *gs*, stomatal conductance; *E*, transpiration; *F*_v_/*F*_m_, maximum quantum efficiency of PSII photochemistry; Ψ_Pd_, predawn water potential; and RWC_Pd_, predawn relative water content.

The percentage of variance explained by each PC is also shown.

Bold values indicate a significant correlation between the variable and PCs.

The two‐way ANOVA showed that the factor *Pr* was significant only in CM (Table 2) even if the significant effect was not due to differences between provenances subjected to the same treatments. This was also true for the significant interaction Pr*WT observed for CE and CS. Overall, as expected, *WT* accounted for most of the variance (Table 2).

**Table 2:** Results of the two‐way ANOVA (*F* and *p* values) for the first principal component (PC1) extracted per each species (see Table 1)

Species	Response variable	Factors	*F* value	*p* Value
CE	PC1	*Pr*	1.58	.232
		*WT*	76.17	**1.50E‐06**
		Pr*WT	9.04	**.011**
CS	PC1	*Pr*	1.78	.196
		*WT*	205.28	**1.20E‐12**
		Pr*WT	6.79	**.016**
CM	PC1	*Pr*	1.58	.232
		*WT*	76.17	**1.50E‐06**
		Pr*WT	9.04	**.011**

The main effects of provenance (*Pr*), water treatment (*WT*), and the first‐order interaction (Pr*WT) are shown. Bold values indicate *p* value <.05. Species are labeled as in Table 1.

No significant differences in mean PI were found between provenances during stress as well as during recovery. Accordingly, PI_stress_ and PI_recovery_ showed roughly the same magnitude (Table 3).

**Table 3:** Phenotypic plasticity index measured in response to stress (PI_stress_) and recovery (PI_recovery_) measured per each considered variable for *Cistus monspeliensis from* southwest Spain (CMSp) and from Sardinia (CMS), *C. salvifolius* from southwest Spain (CSSp) and from Sardinia (CSS), and *C. creticus* subsp. *eriocephalus* from Sardinia (CES) and from the Italian mainland (CEF)

	PI_stress_	PI_recovery_	PI_stress_	PI_recovery_
	CES		CEF	
* A*_a_	0.75	0.60	0.91	0.82
* gs*	0.77	0.54	0.86	0.89
* E*	0.73	0.58	0.90	0.91
* F*_v_/*F*_m_	0.08	0.02	0.07	0.03
* *Ψ_Pd_	0.62	0.42	0.58	0.32
* *RWC_Pd_	0.33	0.28	0.20	0.21
* *Mean	0.55	0.41	0.59	0.53
	CSSp		CSS	
* A*_a_	0.71	0.72	0.63	0.80
* gs*	0.54	0.70	0.61	0.89
* E*	0.48	0.63	0.32	0.83
* F*_v_/*F*_m_	0.45	0.03	0.45	0.04
* *Ψ_Pd_	0.58	0.40	0.63	0.53
* *RWC_Pd_	0.18	0.16	0.21	0.14
* *Mean	0.49	0.44	0.48	0.54
	CMSp		CMS	
* A*_a_	0.71	0.71	0.69	0.72
* gs*	0.73	0.75	0.72	0.79
* E*	0.60	0.66	0.78	0.74
* F*_v_/*F*_m_	0.27	0.07	0.27	0.07
* *Ψ_Pd_	0.43	0.23	0.45	0.45
* *RWC_Pd_	0.11	0.09	0.23	0.26
* *Mean	0.48	0.42	0.52	0.51

*A*_a_, net photosynthesis per unit leaf area; *gs*, stomatal conductance; *E*, transpiration; *F*_v_/*F*_m_, maximum quantum efficiency of PSII photochemistry; Ψ_Pd_, predawn water potential; and RWC_Pd_, predawn relative water content.

Mean values are also shown.

**Figure 2:**
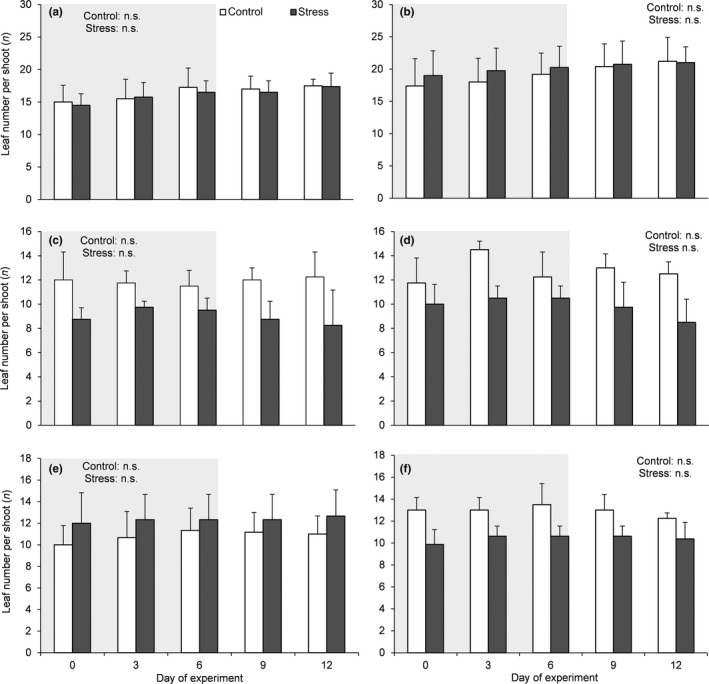
Mean values (±*SD*) of leaf number per shoot in: (a–b) *Cistus monspeliensis* from Sardinia and from southwest Spain, (c–d) *C. creticus* subsp. *eriocephalus* from Sardinia and from the Italian mainland, and (e–f) *C. salvifolius* from southwest Spain and from Sardinia. Differences were considered only within treatment (Control and Stress, white and gray bars, respectively) by considering sampling day as main factor (one‐way ANOVA, *p* ≤ .05, *n* = 10). Shaded areas indicate the days of withholding irrigation

**Figure 3:**
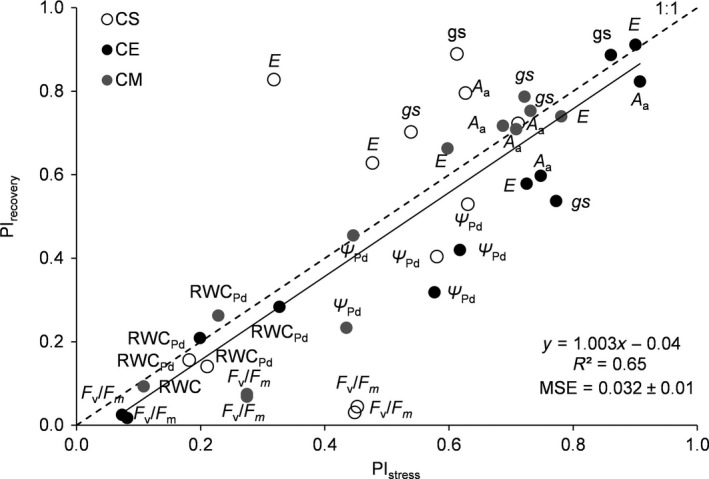
Relationship between the phenotypic plasticity index measured in response to stress (PI_stress_) and that to recovery (PI_recovery_) for the considered physiological traits. Values per each physiological parameter and provenance were used as experimental units (*n* = 36). The fitted equation, its *R* ^2^, the 1:1 line as well as the mean square error (MSE) obtained after cross‐validation (see Materials and Methods section) are shown. *A*_a_, net photosynthesis per unit leaf area; *gs*, stomatal conductance; *E*, transpiration; *F*_v_/*F*_m_, maximum quantum efficiency of PSII photochemistry; Ψ_Pd_, predawn water potential; and RWC_Pd_, predawn relative water content

The absence of leaf shedding was observed in both control and stressed plants (Figure 2) with no significant differences throughout the experiment. A highly significant and positive relationship between PI_stress_ and PI_recovery_ across species was found, and the mean square error (MSE) obtained after cross‐validation was 0.03 ± 0.01 (Figure 3).

## 4 DISCUSSION

A relatively high short‐term plasticity was highlighted in the three species independent of the provenance. As such, the investigated provenances show a similar magnitude of decrease in the physiological leaf traits in response to drought as well as a similar increase in reaching the maximum recovery. The similar plastic behavior between provenances may be explained by considering that the physiological adjustments allow species to cope with short‐term changes in environmental conditions (Bongers et al., 2017; Gratani et al., 2014; Puglielli, et al., 2015; Puglielli, Redondo‐Gómez, et al., 2017 Sultan, 2000). The observed pattern of plasticity was not otherwise necessarily linked to a similar drought response. In fact, the three species show species‐specific strategies in drought response. The relationships Ψ_Pd_ versus *gs*, and RWC_Pd_ highlight that CE and CM are characterized by a greater stomatal control of the photosynthetic process (i.e., higher dependency of *gs* on Ψ_Pd_ changes, Flexas, Escalona, & Medrano, 1998), whereas CS strategy could rely to a greater extent on osmotic adjustments (i.e., lower dependency of RWC_Pd_ on Ψ_Pd_ changes), according to Catoni, Gratani, Bracco, and Granata (2017).

Phenotypic plasticity has often been assumed to be beneficial under stress conditions (see Gratani, 2014 for a review), even if canalization often occurs at the expense of phenotypic plasticity in stressful environments (Valladares et al., 2002). The convergence in terms of the magnitude of phenotypic plasticity found between these congeneric species as well as between provenances in response to drought seems to integrate both views. The results are also in accordance with the ecology of *Cistus* spp. whose ability to counteract stress factors is constrained by their phenology (Correia & Ascensão, 2017). In fact, *Cistus* spp. are relatively short‐lived species that tend to optimize resource acquisition by minimizing construction costs (Hernandez, Pausas, & Vilagrosa, 2011; Lloret, et al., 2016; Puglielli, Cuevas Román, et al., 2017; Saura‐Mas & Lloret, 2007). As a consequence, they would rely on physiological plasticity more than morphological in order to counteract short‐term stressful events. This statement is supported by the greater physiological than morphological plasticity found by Zunzunegui et al. (2009) for the semideciduous *Halimium halimifolium* in response to stress factors. A widely recognized characteristic of *Cistus* spp. is also their resilience ability (Correia & Ascensão, 2017; Gallé et al., 2011; Puglielli, Cuevas Román, et al., 2017) as they can maximize growth (Correia & Ascensão, 2017; Puglielli, Spoletini, et al., 2017) and improve their performance as soon as the favorable conditions are restored (Bongers et al., 2017; Puglielli, Redondo‐Gómez, et al., 2017; Puglielli, Cuevas Román, et al., 2017). A high degree of phenotypic plasticity has often been associated with strong performance under favorable conditions (Lortie & Aarssen, 1996; Valladares, Gianoli, & Gómez, 2007). However, the latter was recently related to a lower survival under drought for *C. salvifolius* (Bongers et al., 2017). This evidence suggests that there is an intrinsic cost deriving from the performance maximization during a favorable period and the ability of the species to face, or even survive, drought. Moreover, this is also in accordance with the results of the meta‐analysis carried out by Zhang et al. (2017). Our results partly contradict these evidences in terms of physiological plasticity patterns in the short‐term. We found a strong positive relationship between PI_stress_ and PI_recovery_ with most of the points falling in the top right part or tightly around the 1:1 line in Figure 3, underlining that a trade‐off exists between the plastic response to stress and that to recovery. The only exception is represented by the combination PI_stress_–PI_recovery_ found for gas exchange parameters in CS, which crossed the 1:1 line. We argue that such a response is related to the lower degree of stomatal control found for CS (i.e., a lower dependency of *gs* on Ψ_Pd_ changes), which favors a high degree of recovery for gas exchange parameters when favorable conditions are restored. In fact, a lower stomatal control of the photosynthetic process in CS, compared to CE and CM, was also found to allow recovery during the transition between winter (i.e., chilling stress) and spring seasons (Puglielli, Cuevas Román, et al., 2017) and it was also reported by Correia and Catarino (1994) in response to drought. We can affirm that CS falls in such trade‐off.

Our results support the conclusion that the highlighted trade‐off can justify the costs of the greater plasticity in response to stress generally found for *Cistus* spp. (Bongers et al., 2017; Catoni et al., 2012; Grant et al., 2014). Also, the absence of leaf shedding during stress suggests that the observed trade‐off may be involved in delaying leaf shedding during short‐term stress periods, thus providing a profit for these species. These findings fit the ecological behavior of such species and call for further research on the physiological mechanisms that allow such responses. We argue that one of the mechanisms behind this trade‐off is represented by leaf paraheliotropism, a key aspect of the adaptive strategies of *Cistus* spp. (Correia & Ascensão, 2017; Flexas et al., 2014; Gratani & Bombelli, 1999; Oliveira & Peñuelas, 2004; Werner, Correia, & Beyschlag, 1999, 2002), which are known to favor gas exchange recovery in these species after drought stress suppression (Puglielli, Redondo‐Gómez, et al., 2017). However, the underlying mechanisms of such response are beyond the scope of the present work.

In terms of the considered parameters, *F*_v_/*F*_m_, a measure of the degree of photoinhibition (Oliveira & Peñuelas, 2002), was the only one which was decoupled from the PI_stress_–PI_recovery_ trade‐off. This is not surprising, as both physiological and morphological mechanisms are known to be less effective in allowing *F*_v_/*F*_m_ recovery after drought stress in *Cistus* spp. (Correia & Ascensão, 2017). Otherwise, the results suggest that changes in *F*_v_/*F*_m_ do not represent a major factor in drought stress‐induced photosynthesis depression as gas exchange parameters widely recovered after restoring irrigation, according to the result obtained for other Mediterranean species (Catoni et al., 2017; Flexas et al., 1998). Moreover, low Ψ_Pd_ does not always lead to severe reduction of *F*_v_/*F*_m_ in *Cistus* leaves (Grant et al., 2014). We suggest that *F*_v_/*F*_m_ response may be mostly related to very long‐term drought events in these species. However, as *F*_v_/*F*_m_ changes in *Cistus* spp. are also buffered by structural modifications of the light‐harvesting complexes in leaves formed in different environmental conditions (Grant et al., 2014; Puglielli, Cuevas Román, et al., 2017), *F*_v_/*F*_m_ interpretation can be much more complex. At any rate, our statements on the absence of a significant photoinhibition are confirmed by the lack of leaf shedding in stressed plants. Leaf shedding during summer in *Cistus* spp. is in fact caused by chronic photoinhibition, which is usually detected through deep *F*_v_/*F*_m_ reduction (Werner et al., 1999, 2002).

In conclusion, our results confirmed the hypothesized trade‐off between the extent of the plastic response to stress and that to recovery. The trade‐off contributes in explaining the profit, more than the cost, of a higher physiological plasticity in response to short‐term stress imposition for *Cistus* spp. Based on our evidence, the highlighted behavior is involved in avoiding premature leaf shedding in short‐term stressful events. This may also represent a good strategy to hold the more costly summer leaves (compared to winter leaves, Puglielli G. & Varone L. unpublished) in order to maintain relatively high productivity to face more prolonged drought events. Species that are constrained to maximize their performance during the favorable period, such as *Cistus* spp., have to sustain a higher cost in terms of plasticity when drought occurs. We demonstrated that such cost is paid back in terms of a higher short‐term plasticity in the recovery.

However, the relationship PI_stress_ versus PI_recovery_ seems to be parameter‐specific and therefore deserves to be tested including a wide range of species and traits in order to get a deeper overview of the costs and/or profits arising from a greater plasticity in response to stress factors. This is particularly important considering that climate change will modify the frequency of stressful events (Giorgi & Lionello, 2008), calling for major attention on the resilience mechanisms of species inhabiting environments threatened by climate change, such as the Mediterranean Basin.

## CONFLICT OF INTEREST

None declared.

## AUTHOR CONTRIBUTIONS

All authors provided substantial contributions to conception and design, acquisition of data, or analysis and interpretation of data, drafted the article and revised it critically for important intellectual content, and gave final approval of the version to be published.
